# EARLY CHARACTERISTICS AND BIOMARKERS OF COGNITIVE IMPAIRMENT AND DEMENTIA WITH PRIOR MOTOR IMPAIRMENT

**DOI:** 10.1093/geroni/igac059.1313

**Published:** 2022-12-20

**Authors:** Qu Tian, Susan Resnick, Luigi Ferrucci

**Affiliations:** National Institutes on Aging, Baltimore, Maryland, United States; National Institute on Aging, Baltimore, Maryland, United States; National Institute on Aging, Baltimore, Maryland, United States

## Abstract

Motor impairment is one early feature of preclinical Alzheimer’s disease and strongly predicts future dementia. Whether older persons with dementia who had prior motor impairment present specific characteristics is less clear. In the Baltimore Longitudinal Study of Aging, we compared characteristics of participants with cognitive impairment or dementia who had slow gait on average 6 years before symptom onset (<1.0m/sec)(n=65,81±7 years,35%women,25%black) to those who did not (n=93,77±7 years,61%women,16%black). Early cognition and key biomarkers on average 7 years before onset were compared between groups using multivariable linear regression, adjusted for demographics. Those with prior slow gait had early unfavorable metabolic panel (higher fasting glucose,higher 2hr-OGTT insulin,lower HDL,higher triglyceride,higher BMI and waist,higher blood pressure), lower manual dexterity and processing speed, and lower lysophosphatidylcholine 18:1 and 18:2, critical in the synthesis of cardiolipin-an essential component of mitochondrial membranes. Strategies to prevent metabolic dysfunction at an early stage may slow down dementia progression.

